# Taming Prebiotic Chemistry: The Role of Heterogeneous and Interfacial Catalysis in the Emergence of a Prebiotic Catalytic/Information Polymer System

**DOI:** 10.3390/life6040040

**Published:** 2016-11-04

**Authors:** Pierre-Alain Monnard

**Affiliations:** Department of Physics, Chemistry and Pharmacy, University of Southern Denmark, Campusvej 55, DK-5230 Odense, Denmark; monnard@sdu.dk; Tel.: +45-6550-4437; Fax: +45-6615-8760

**Keywords:** RNA world, non-enzymatic polymerization, RNA catalysis, heterogeneous catalysis, self-assembled and self-organized media

## Abstract

Cellular life is based on interacting polymer networks that serve as catalysts, genetic information and structural molecules. The complexity of the DNA, RNA and protein biochemistry suggests that it must have been preceded by simpler systems. The RNA world hypothesis proposes RNA as the prime candidate for such a primal system. Even though this proposition has gained currency, its investigations have highlighted several challenges with respect to bulk aqueous media: (1) the synthesis of RNA monomers is difficult; (2) efficient pathways for monomer polymerization into functional RNAs and their subsequent, sequence-specific replication remain elusive; and (3) the evolution of the RNA function towards cellular metabolism in isolation is questionable in view of the chemical mixtures expected on the early Earth. This review will address the question of the possible roles of heterogeneous media and catalysis as drivers for the emergence of RNA-based polymer networks. We will show that this approach to non-enzymatic polymerizations of RNA from monomers and RNA evolution cannot only solve some issues encountered during reactions in bulk aqueous solutions, but may also explain the co-emergence of the various polymers indispensable for life in complex mixtures and their organization into primitive networks.

## 1. Introduction

Prebiotic chemistry is intimately linked with the emergence of life and should therefore have comprised most chemical processes that yielded biomolecules or at least their precursors relevant for the formation of precellular entities. It also determines at least in part the types of molecules that compose cells even today. Although the chemical definition of life is still debated, the ubiquitous presence of biopolymers at the core of the metabolism (peptides), information make-up (DNA, RNA) of cells and that of other molecule types (lipids for membranes) highlight the need for their early development, perhaps already during the time period in the Earth history that coincides with prebiotic chemistry. However, depending on the approach to that question, whether one decides to explore a top-down or a bottom-up perspective, one will be confronted with different questions that pertain to the core of prebiotic chemistry. The top-down approach is defined as a retrospective search that looks at common attributes of contemporary cells that likely were already present in the last common ancestor(s) (LCA) and might have been present at the origin of life. That is, the time interval between the emergence of the first “living” systems and the LCA, as well as the likely large difference in the organization of these systems leave the door open for intermediate systems, products and potentially victims of evolutionary processes, which might have left no traces in contemporary biochemistry.

Considering the bottom-up approach, a prospective process, the main issue lies in the availability of the compounds on the prebiotic Earth, which is linked to the geochemical conditions on the early Earth. Unfortunately, the dearth of geological evidence on this period prevents us from having a clear picture. Indeed, this approach is clouded by uncertainties about the sources of prebiotic chemicals, which would depend on the flux and nature of extraterrestrial compounds delivered on the Earth and the nature of the primitive environment (atmosphere, ocean(s) and possibly landmass(es)). Even in the event that the chemical molecules might have existed, their reactivity (stability, propensity to react, e.g., polymerize or decompose) in this environment would have further defined whether they could accumulate, polymerize and later whether the level of chemical complexity required for the emergence of life could be attained.

## 2. RNA World Hypothesis

The RNA world hypothesis postulates that modern life, based on DNA, RNA and proteins, was preceded by a primitive form of life in which RNA replaced both DNA and proteins, as a genetic and catalytic polymer, respectively. Its prebiotic synthesis is therefore one of the most important steps in chemical evolution. [1,2,3] This idea is supported by the discovery of RNA catalysts [4,5] and the presence of “molecular” fossils in contemporary cells. For instance, the RNA catalysis of the peptidyl bond formation in the ribosome [6], the ubiquity of metabolic co-factors that are composed of a ribonucleotide moiety or have functional groups that resemble the structure of nucleotides and the synthesis of deoxynucleotides proceeding via ribonucleotide intermediates [7].

The fundamental requirements of the RNA world hypothesis are (i) monomer (ribonucleotides) availability; (ii) efficient non-enzymatic spontaneous and template-directed polymerizations, that is, reactions whose RNA production must have at least outpaced the degradation of their products; (iii) the capacity of short non-enzymatically polymerized RNA oligomers to possess a catalytic/binding function and (iv) their capacity to self-assemble into long strands capable of evolved catalysis. These various stringent requirements have led some of the pioneers in the field to qualify the RNA world hypothesis as a “biochemist dream.” [1].

### 2.1. Availability of the Ribonucleotides

**Figure 1:**
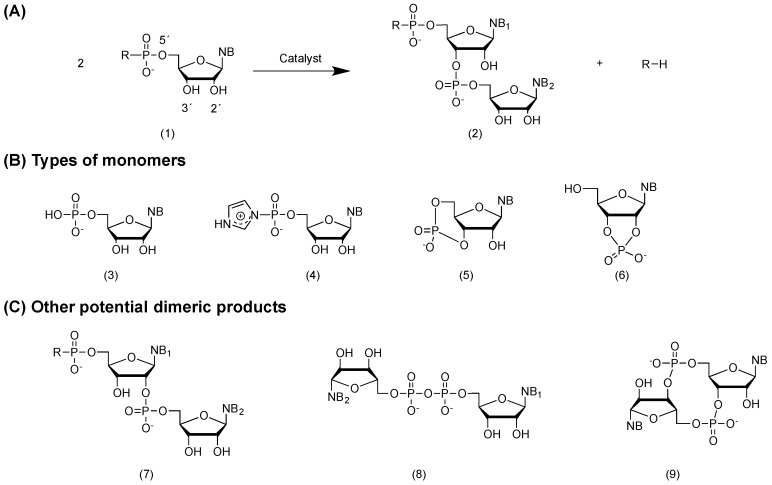
Chemical structures and reactivity of RNA monomers. (**A**) General reaction scheme. Two monomers (**1**) react in the presence of a catalyst, e.g., metal-ions, amines, peptides or heat, to form a dimer (**2**) releasing a by-product R-H. The bond formed is a phosphodiester bond, but other types of chemistry have been proposed [17]; (**B**) Types of monomers proposed: (**3**) ribonucleotide monophosphate; (**4**) ribonucleotide monophosphate imidazolides, here imidazole (Im); many derivatives of imidazoles or other chemical activation groups have been used [17]; (**5**) 3′,5′ cyclic ribonucleotide monophosphate; (**6**) 2′,3′ cyclic ribonucleotide monophosphate. The by-product R-H is water when monomers (**3**), (**5**) and (**6**) react and an imidazole with monomer 4; (**C**) Other potential dimers: (**7**) 2′-5′ dimer, (**8**) 5′-5′ pyrophosphate dimer and (**9**) 3′-5′ cyclic dimer. The types of predominant products obtained during polymerization depend on the nature of the monomer (nature of the nucleobase (NB) and type of activation).

The availability of the ribonucleotides has long been an unresolved issue. RNA monomers are conjugates of three different chemical parts, a phosphate, a ribose and a nucleobase (Figure 1A). One synthetic difficulty encountered is more related to the linking of the parts into the monomers, especially the glycosidic bond formation, than to the synthesis of each part individually. Indeed, the synthesis of nucleobases from hydrogen cyanide or mixtures of formamide and urea, most recently in interstellar ice models [8], that of sugar by the formose reaction [9] or the availability of phosphate through leaching of phosphorus from Fe/Ni-rich meteorites [10] have been demonstrated. However, an efficient way to join these parts into a nucleotide remains elusive. Taking a different approach based on the use of prebiotically-plausible small substrates, Sutherland and co-workers, however, managed to synthesize pyrimidine nucleotides in a series of synthetic steps, even though the reaction conditions are significantly altered between each step, and the ordered addition of reactants is necessary [11].

It should also be noted here that as for any prebiotically-relevant species, a multitude of related, but different compounds would have likely been “by-products” of prebiotic syntheses as exemplified by the formose reaction that only delivers a small amount of ribose [9] among a multitude of sugars or by the nucleobase synthesis from formamide and urea [8] that yields numerous heterocycles besides the extant nucleobases. The presence of “by-products” might have either promoted the formation of the ribonucleotides, or their polymers or alternatively inhibited it, or even lead to the decomposition of the “target” molecules. The rapid decomposition of nucleobases [12] and sugars [13] in aqueous solution also questions the concept of the RNA world. All of these observations have led some researchers to doubt the prebiotic assembly of “pristine” RNA, as the first ancestral information molecules [14,15,16]. In fact, the properties of RNA and their perfect suitability for current RNA functions suggest that RNA may be “an emergent, highly refined molecule” [16].

### 2.2. General Consideration on Non-Enzymatic RNA Polymerization from Monomers

**Figure 2:**
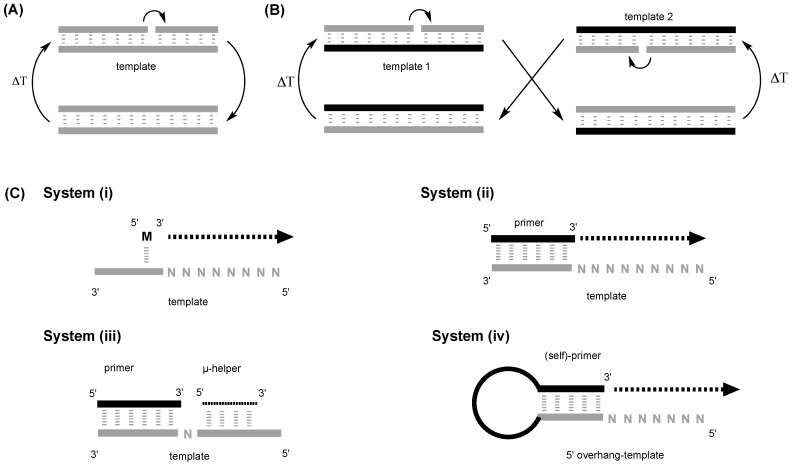
Template-directed polymerizations. (**A**) Self-replication versus (**B**) cross-replication. Self-replication was the first successful replication reported [21] and is based on the fact that the copying of a template sequence leads to its direct amplification. Cross-replication of nucleic acids is similar to biological information replication: a template sequence is first copied given a RNA fragment that is complementary to the original sequence. This new fragment can then act as a template and be itself copied, yielding the original sequence. (**C**) Schematic representation of the various systems used in the exploration of template-directed (TD) polymerization (N denotes a templating residue on the template): (i) monomer/template. In this system, monomers have to associate, and they will condense in the 5′ to 3′ direction (arrow); (ii) primer/template/monomer. The primer and the template hybridize and the monomers will associate with the template while stacking at the end of the primer. They will condense in the 5′ to 3′ direction (arrow) (iii) primer/template/monomer and micro-helper oligomers. In this situation, only one monomer can be added at a time, and the micro-helper has to be exchanged for a new round of polymerization to start; and (iv) hairpins. The 5′-end of the hairpin is the templating sequence and the 3′-end the primer. The length of the stem in the number of base pairs will determine the strength of hybridization. The condensation will occur as described for System (ii).

Assuming that RNA monomers were available, the next steps would be their incorporation into oligomers and polymers relying on their chemical reactivity and simple catalysts, as enzymes were absent on the early Earth. When considering this type of polymerization, one should differentiate between monomer self-condensation and template-directed (TD) polymerization (Figure 2). In the former reaction, nucleotides need to aggregate by themselves into supra-molecular assemblies, such as extended monomer stacks, before being linked together into nucleic acid polymers. The resulting molecules can then either be further elongated by additional monomers or be ligated together to form ever-longer molecules. In the latter reaction, the nucleotides assemble onto a template (i.e., a pre-existing nucleic acid polymer) by molecular recognition, a process involving a complex interplay of dynamic intermolecular interactions. The template sequence, i.e., the nucleobase order on the template, should ideally determine the nucleobase sequence of the polymeric product via Watson–Crick base pairing. Thus, a sequence transfer should occur between the template and the newly-synthesized polymer. This process should allow for the amplification of nucleobase sequences and is also called replication. Two different types of replication can formally occur: self-replication and cross-replication in the case of palindromic and non-palindromic template sequences (Figure 2A,B). The challenge of the prebiotic replication is to achieve such a sequence-specific process in the absence of enzymes and their proof-reading functions, which ensure low error rates in biological organisms, and thereby avoid error rates that would jeopardize the information transmission. Sequence amplification, in particular those exhibiting a catalytic activity, would have been of paramount importance to the emergence of an RNA world [1,18].

In both cases, the formation of the phosphodiester bond between two monomers is likely a bi-molecular nucleophilic substitution (S_2_N) reaction [19], a condensation reaction that produces a molecule of water for each phosphodiester bond formed (Figure 1A). Condensation reactions at low concentrations of monomers are disfavored per se in an aqueous medium, while polymer hydrolysis occurs more readily. In the case of ribonucleotides (5′-monophosphate), the 2′-OH and 3′-OH are competing nucleophiles leading to several possible products (Figure 1A,C). Experiments by, among others, Kanavarioti and coworkers demonstrated that the 2′-OH is the stronger electrophile, and the 2-product is kinetically favored, while the 3′-products are thermodynamically more stable [19]. The formation of RNA phosphodiesters is endothermic with an energy cost that should be similar to that of the DNA phosphodiester bond measured at 5.3 kcal mol^−1^ [20]. To overcome this energy requirement, i.e., reduce the energy barrier, two approaches can be followed: (i) the use of chemical activation and prebiotic catalysts and (ii) that of heat.

Compared to monomer self-condensation, template-directed polymerization is more sensitive to repulsive interactions between charged reacting species during the duplex formation that is necessary for this polymerization type. Each monomeric subunit of oligonucleotide templates contains at neutral pH one negatively-charged phosphate moiety that ensures template solubility. However, these charges need to be shielded by cations for the hybridization with monomers and primers to occur; otherwise, the electrostatic interactions of opposing phosphate groups would destabilize the duplex organization. Thus, these reactions are usually conducted in high-ionic strength media, e.g., using sodium chloride and magnesium chloride, but other molecules, such as polycationic species, could be envisioned.

### 2.3. Chemical Activation of RNA Monomers 

Chemically-activated nucleotides, the nucleotide triphosphates that are used in biological systems, exhibit a very low reactivity as monomers for non-enzymatic polymerization reactions [1]. Other activation groups that are better leaving groups than pyrophosphates [17] have been proposed, e.g., imidazole (Im) phosphoramidates (Figure 1(4)) [22]. This particular activation group type was selected, because it seemed to be relatively plausible under prebiotic conditions and was easy to use to pre-activate nucleotides. The half-life of the activated species in an aqueous solution depends on the chemical identity and the presence of hydrolytic metal ion catalysts, as well as the medium conditions (temperature, pH) [17].

Instead of activating the nucleotide monomers prior to the reaction, it is also possible to use water-soluble in situ coupling reagents, cyanogen, cyanamide or various derivatives of carbodiimide. Such a process might model more accurately the situation on the early Earth and does reduce the number of reaction and purification steps and also resolves the hydrolysis problem observed with pre-activated monomers. The drawback of this approach lies in the number of additional reaction components and the formation of side products that might interfere with the condensation reaction [23]. Water-soluble carbodiimides, like 1-ethyl-3-(3-dimethylaminopropyl)carbodiimide (EDC) [24] and hydroxy-7-azabenzotriazole (HOAt), have been used [25,26,27]. In the laboratory, 10 molar equivalents of EDC for guanine monomers and 50 molar equivalents for thymidine/uridine monomers are however required to yield an efficient coupling [23]. In these investigations, 1-methylimidazole (1-MeIm) was found to be the best additive (better than 2-MeIm) [23]. Others studied in situ activation agents, such as cyanamide [28] and cyanogen [29,30], which seem even more prebiotically plausible [31], which were also shown to be efficient for chemical ligation reactions between nucleic acid oligomers.

Finally, the cyclic forms of nucleotides represent another form of activated nucleotides [32]: the 2′-3′ species (Figure 1(6)), which is the product from the decomposition of RNA oligomers, and the 3′-5′ ones (Figure 1(5)). Both will react under dry or almost dry conditions [33], the 3′-5′ cyclic monomers being more reactive. However, their availability is still being debated due to their high energy and related rapid decomposition [33,34]. All chemical activation techniques can in principle be applied to activate nucleic acid oligomers, as well.

## 3. RNA Polymerization in Bulk Aqueous Media

### 3.1. Non-Enzymatic Self-Condensation of RNA Monomers in Aqueous Media

Even though condensation reactions at low concentrations of monomers are disfavored per se in an aqueous medium, the first investigators erroneously expected the π-π interactions between the hydrophobic heteroaromatic ring structures of the nucleobases (i.e., spatial interactions of nucleobases with neighboring rings through electron fluctuations) to force nucleotides to align into stack-like organizations conducive to polymerization. Indeed, different experiments had shown that base-stacking has the largest contribution to the stability of nucleotide duplexes in aqueous solutions [35]. 

Early investigations were carried out in a rather unsystematic fashion due to the lack of solid information on the geochemical conditions prevalent on the early Earth [17]. Overtime, 5′-phosphoimidazolide derivatives became the main type of monomers used to investigate monomer self-condensation at low monomer concentrations and in conjunction with metal-ions whose catalytic activity significantly improved reaction rates. The condensation efficiency of metal ions can be listed in the following sequence (lowest to highest): Hg^2+^ ≈ Cu^2+^ ≈ none < Mg^2+^ ≈ Ca^2+^ < Cd^2+^ ≈ Fe^2+^ < Ni^2+^ < Mn^2+^ < Zn^2+^ < Co^2+^ < Pb^2+^ [36]. Their catalytic activity roughly corresponds to their affinity for nucleobase residues [37]. The uranyl ion UO_2_^2+^ is also described as an efficient condensation catalyst that favors 2′-5′ regioselectivity [17]. 

The reaction yields in terms of total condensation generally remained low, and short oligomers were the main products in aqueous solution at low monomer concentrations: uridylate being the most difficult monomer to oligomerize. The 5′-capping of the oligomeric products by a pyrophosphate dimer (NppN, Figure 1(8)) and cyclization of oligomers can occur [38]. However, 2′-5′ linkages predominate in the linear oligomeric products. To boost the polymerization yields for imidazolide monomers, Kanavarioti and co-workers increased the monomer concentrations above the 1 M concentration without significantly improving reaction yields or product lengths [39,40]. 

In most cases of successful self-condensation of 3′,5′ cyclic nucleotides (cNMP), the reaction proceeded by drying and/or heating melts, a procedure whose simplicity seemed appealing in the light of the origins of life. Under these conditions, 3′,5′ [41] and 2′,3′ cNMP [42] were reported to condense into penta- and hexa-mers. The hexameric products contained mostly 3′-5′ linkages when aliphatic diamines were added to the reaction mixture (pH ca. 8, 25–85 °C). In more recent reports [33], the 3′,5′ cGMP were polymerized into 40 mers after a 15-h incubation at 50 °C, but the authors estimated the monomer incorporation to be less than 2%. Some residual bulk water completely precluded the polymerization. 

### 3.2. Non-Enzymatic, Template-Directed Polymerization of RNA Monomers in Aqueous Media

A plausible non-enzymatic, template-directed polymerization of ribonucleotides that could occur in the prebiotic environments is necessary to realize the RNA world, but its mechanistic nature is still an open issue. However, the functionality of RNAs extensively depends on their conformation, which is defined by the interactions between secondary and tertiary structure motifs whose assembly is conditioned by the nucleobase sequence and interactions with solutes, e.g., divalent cations. The resulting motifs (hairpins, pseudo-knots) and structures (guanosine quadruplets and triple helices) can partially block the template-directed polymerization, as the interactions between strands are more stable than the interaction of a template and monomers. Studies on the stability of secondary and tertiary structure motifs and on the activity of ribozymes have demonstrated that solutions exist to prevent their formation. One solution is to chelate metal ions that stabilize the RNA in an undesired conformation. Another is to use molecules or surfaces that interact with the ribose-phosphodiester backbones to unfold a nucleic acid molecule.

To explore TD polymerization, various systems have been used: System (i) linear template; (ii) linear template/primer; (iii) linear template/primer with small oligomers (4- to 6-mers) that hybridize downstream from the insertion point and (iv) hairpins, which can be seen as “primers covalently linked to their template” (Figure 2B). In System (i), the formation of a complete, correct copy of the template requires the monomers to align themselves on neighboring template bases and form stacks that fill the whole template. The polymerization in a stack of monomers can occur between any two monomers, which could prevent the full copying of a sequence. Systems (i) and (ii) are very similar as far as the polymerization process and rates are considered: short pre-synthesized oligonucleotides that hybridize with the template (primers) precisely define the starting point of the polymerization or primer elongation (at the 3′ end of the primer). As demonstrated by Kanavarioti and co-workers [43], the formation of a dimer is the rate determining step in the template/monomer System (i), and the rate for the subsequent formation of the trimer is an order of magnitude higher, while the elongation to longer oligomers (≥4-mer) is even faster. Thus, the primer elongation from the 3′/2′ hydroxyl of the primer end will predominate [44]. The hybridization can force the template into an A-helix form for RNA that clearly enhances the rates [45], and the primer offers a base-stacking surface for the new approaching base, which will improve monomer association with the templating sequence. In a few studies [24,25,46], the interactions between the incoming monomer and template at the 3′ end of the primer were strengthened by the addition of short oligomers (4 to 6 nt) that hybridized downstream from the monomer insertion point (System (iii)): in such a system, the incoming monomer is associated more tightly with the template due to the increased stacking, hence increasing its probability to be linked to the primer. However, this last system should be viewed as a technological fix to improve sequence-specific monomer incorporation than a truly plausible prebiotic setup. System (iv) should exhibit a more efficient (because of its intramolecular character) hybridization between template and primer than in the previous tri-component system. In addition, the hybridization can be reinforced by the loop sequences, e.g., UUCG or GCAA [47].

In contrast to cellular replication, the templates used in non-enzymatic template-directed polymerization that are considered here are relatively short. Thus, the reaction efficiency in terms of monomer incorporation and the length of the products will be influenced by the reduced monomer stacking observed at both ends of a template [48,49], even in the case of primer elongation [50,51], a fact that will affect the completion of replication if the template ends directly at the end of the templating sequence.

The experiments aiming at uncovering the intricacies of the non-enzymatic TD polymerization were usually conducted using imidazolide monomers, metal-ion catalysts, such as Mg^2+^, Pb^2+^, Zn^2+^, [UO_2_]^2+^, or mixtures thereof in high-ionic strength media (monovalent salts for duplex stabilization) and at low temperatures (0 °C to 4 °C) to enhance the intermolecular interactions between monomers and templates, as well as primers in the corresponding studies. Using a template/monomer system, the insertion of activated monomers on the homopolymeric templates with cognate residues only yielded good incorporations of activated G monomers on poly(C) templates [52,53] with products of a monomer unit length in excess of 40. Dependent on the identity of the activation group, the reaction could be extremely regioselective, as products obtained from 2-methylimidazole pG (2-MeImpG) were exclusively 3′-5′ linked [54]. Polymerization yields and rates decreased in the following sequence: G >> A > C > U, on their respective homopolymeric templates.

In experiments with heteropolymeric templates (template monomer system) and the hairpin/monomer system, the product yields heavily depended on the template sequences and template length. In these systems, the major obstacle to an efficient template-directed reaction was again the poor reactivity of activated uridine [44,50,55,56,57,58,59], even if U derivatives were used and D nucleobases replaced A (three H-bonds can be formed by D•U). In fact, activated G and C nucleotides could only polymerize efficiently, if the poly(G,C) template sequence contained over 60% of C-residues [1]. If the template sequence contained more than one other residue (A, G, U) between stretches of C residues, replication became extremely slow in the case of GT or TG sequences or was even completely blocked by AT, TA, AA, GA and AG sequences [50,51,59]. Note that DNA templates were often used in the laboratory, as their decomposition is negligible. In a template/primer/monomer system [60], the elongation of the primer was also inhibited by mixtures of monomers (monomer cross-inhibition), and the elongated products often contained mismatches. In particular, G monomers were added instead of D/A monomers possibly due to the formation of a wobble base pair between a U residue on the template and activated G monomers.

In general, the regioselectivity of the phosphodiester bonds depended on both the catalyst identity and the activation group of the monomers. Even though a relatively efficient template-directed polymerization on heteropolymeric templates can be achieved, the main obstacle for a true amplification of RNA in bulk aqueous solution remains the fact that a RNA product obtained on a template containing 60% C residues will itself never be a template for cross-replication, as it contains only 40% C residues.

In an attempt to alleviate these issues (U incorporation and related slow polymerization rates) encountered in the non-enzymatic TD polymerization in bulk aqueous media, experiments with monomers with chemical modification either on their nucleobases or their sugar were carried out. From the point of view of the origins of life, the use of non-canonical nucleobases may be extremely relevant, as it is likely that other non-canonical nucleobase were formed simultaneously to the canonical ones [15]. The replacement of the phosphodiester bonds or even the phosphate-ribose backbones has been proposed to solve the difficulties inherent to the synthesis of RNA from a chemical point of view [16,61]. However, the potential to interact with RNA should not be lost to ensure evolutionary continuity.

Improvements of the polymerization rates of A could only be obtained by replacing the weak base pair A•U by D•5′-propynyl-U derivatives whose base pair possesses an additional H-bond [57] or using the 2-thiouridine derivative (2-thioU) [62]. In the latter case, the use of 2-thioU in the template did not improve the incorporation yields of activated adenosine monophosphate (AMP), except when the templating sequence contained a single 2-thioU and otherwise multiple C residues.

The replacement of one of the ribose hydroxyl groups by an amino group increased the polymerization rates as expected from the higher nucleophilicity of the amino group [63]. Template-directed polymerization of 3′-amino-3′-deoxyribonucleotides proceeded more efficiently, but the regioselectivity of the products was not improved per se, as a significant number of 2′-5′ phosphodiester bonds were also formed. Activated, 2′- or 3′-amino-2′- or 3′-deoxyribonucleotides (2′-NH_2_-2′dNMP or 3′-NH_2_-3′dNMP), 2′- or 3′-amino-2′,3′-dideoxynucleotides (2′-NH_2_-ddNMP or 3′-NH_2_-ddNMP) could be better incorporated into products than activated thymidine monophosphate (TMP) or deoxyuridine monophosphate (dUMP).

The use of these activated amino DNA monomers or 2-thioU has also permitted the discovery of an inherent property of the non-enzymatic polymerization that clearly supports the possibility of an efficient propagation of the genetic information in a non-enzymatic fashion. It was long considered that one major drawback of this type of polymerization was its error-proneness (close to 20% of misincorporation) [64], which would extremely reduce the applicability of this method for a nucleic acid of a length corresponding to known enzymatically-active RNA molecules. Irene Chen and co-workers [65,66] have however shown that the incorporation of a noncognate nucleobase at the 3′-end of a primer (terminated with 3′-NH_2_-ddNMP) reduces rates of a subsequent elongation by more than two orders of magnitude compared to those observed in the case of correct elongation. This phenomenon was coined stalling and has been recently confirmed with 2-thioU [62]. Thus, the intrinsic dynamics of the reaction could well steer the nucleic acid production towards sequence fidelity higher than expected from its inherent error rates.

While both monomer self-condensation and non-enzymatic TD polymerization in bulk aqueous solutions have broadened our understanding of RNA polymerization, it is clear that a number of issues remain: in particular, (i) the uridine derivative lack of reactivity; (ii) the fact that RNA polymerization in bulk aqueous solution was always conducted at high monomer and catalyst concentrations and in isolation, i.e., a relative pure system in terms of bystander molecules or other chemical systems; and (iii) the link between RNA products and their potential activity is still elusive. This last issue relates both to the chemical nature of the RNA products (e.g., regioselectivity of phosphodiester bond and molecule length in monomeric units) and type of activity that might be expected as the primitive RNA function.

## 4. Heterogeneous Media as Supporting Environments for RNA Polymerization

### 4.1. Rationales behind the Heterogeneous Media and Their Nature

Bulk aqueous solutions are not conducive to the extensive condensation and TD polymerization reactions. Reactants are diluted very quickly, as diffusion rates are high, and water shifts the equilibrium towards hydrolysis. RNA polymerization reactions usually require critical concentrations of monomers [40] or of their precursors [24] in the range of several 10 s to 100 s mM [17] for an effective product synthesis (both in terms of numbers of molecules and their length). Such concentrations on the early Earth seem to be unrealistic in a bulk aqueous medium. To overcome this issue, adsorption on mineral structures, i.e., the recourse to heterogeneous catalysis, was proposed early on [67]. This view has gained traction, and a number of authors are now advocating the study of integrated molecular systems and of their emergence within putative geological environments [68].

The type(s) of heterogeneous media that would be available depends on geochemical conditions on early Earth. As mentioned earlier, to definitely ascertain them is difficult given the evidence that is available. Researchers made very different propositions about the conditions prevalent on the early Earth: hot/cold (even mostly ice-covered Earth), completely covered by water or with proto-continents, i.e., with a solid crust and the potential for fresh water. The chemical compositions of aqueous/atmospheric environments are also debated. It is also plausible that the early Earth was a patchwork of microenvironments. The exploration of the universe in search for planets and planetoids capable of sustaining life undertaken in recent years has also established that (i) most water in the universe is present as ice; (ii) ice-covered celestial bodies, e.g., Europa and Enceladus in our solar system, have liquid water below their ice sheets and (iii) many stars possess planets that could harbor water.

**Figure 3:**
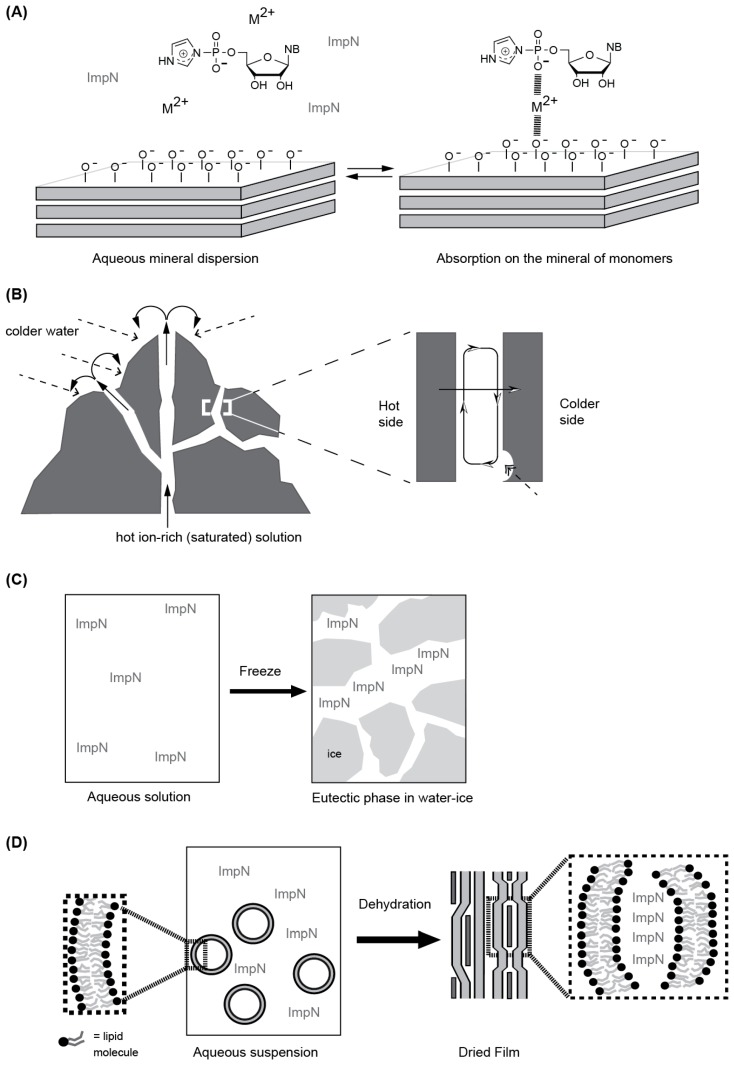
Schematic representation of the heterogeneous media. (**A**) Mineral surfaces. For example, clays, such as montmorillonites (the most studied ones), consist of alternating tetrahedral and octahedral silicate layers. Their surface possesses easily exchangeable metal ions, whose replacement defines the surface charge [69]. During weathering, negative charges appear that can interact with the activated ribonucleotides and oligomers. (**B**) Channels in mineral formations. When hot aqueous solutions containing high concentrations of inorganic ions come in contact with colder water, e.g., on the sea floor [70], the ions precipitate in solid formation around the jet of hot water (arrow with hashed tail). When a temperature gradient exists in the resulting water channels, processes called thermal convection and thermophoresis (half-white head arrows in the insert) can take place that can in conjunction with gravity concentrate solutes, both ribonucleotides [71] and oligomers [72], present in the aqueous solution, especially if a depression in the rock can serve as a trap (double-head arrow with hashed tail). (**C**) Eutectic phase in water/ice. This heterogeneous system is formed upon lowering the temperature of a reaction sample containing ionic solutes past its freezing point. At that temperature, water begins to nucleate and form pure water ice, which effectively reduces the volume of the remaining liquid that forms a network of brines (channels containing liquid water) [73,74]. (**D**) Organic molecule aggregates. Organics, such as amphiphiles, peptides and sugars, can self-assemble into organic structures that can compartmentalize solutes. In the case of closed lipid bilayers, so-called liposomes, the bilayers structures themselves will serve during dehydration processes, and solutes present around/within the structures will be encapsulated in the bilayer fragments that are deposited on a support. The resulting mixed layered aggregates are not in a solid, but rather a liquid crystalline state [75].

Thus, depending on the researchers’ assumptions, several types of heterogeneous polymerization environments can be proposed: mineral formations and surfaces, eutectic phases in water/ice and water/salt systems and self-assembled molecular systems, such as lipid aggregates (Figure 3). Several examples of these heterogeneous environments have been investigated for their ability to promote or catalyze RNA polymerization reactions, as well as the activity of functional RNAs (see below).

In general, the heterogeneous catalytic environments are expected to fulfill at least some of the following functions: concentrate reactants (monomers, template, catalysts), provide a micro-environment (i.e., a form of compartmentalization) that is conducive to RNA polymerization, i.e., an environment where the reactants are to some extent ordered into molecular configurations promoting the reaction and reduce the hydrolysis of both products and activated reactants. Additional functions could also be expected, such as the selection of the ribonucleotide monomers or their molecular precursors from other bystander molecules and support of the RNA product activity, as well as perhaps favoring specific RNA linkage regioselectivity or, in general, specific chirality.

#### 4.1.1. Mineral Surfaces and Formations

Minerals have been considered as catalytic surfaces [67] or micro-environments that would permit the accumulation of solutes, even in a selective manner either via specific direct association of solutes on the mineral surface (Figure 3A) or by allowing accumulation of reactants by thermophoresis and convection processes in channels or small pores (Figure 3B) within the mineral formation [71].

In the case of direct association, the absorption should permit the accumulation of ribonucleotides, position the molecules in reactive conformation and protect them from degradation as proven by, e.g., the interactions of silica with ribose, which promote its ribofuranose form and prevent its degradation [76], or the stabilizing interaction of borate with ribose [77]. The catalysis of RNA monomer self-condensation and TD polymerization has been evidenced on several minerals [17], most prominently on clays, such as homoionic montmorillonites. In a seminal article [78], Ferris et al. demonstrated the synthesis of RNA of up to 50 monomeric units in length in the presence of either a short nucleic acid oligomer (DNA with one ribonucleotide at the 3′-end) or pyrophosphate dimer (RNA), as condensation initiators. Fresh activated monomers were regularly added to counteract the hydrolysis of the monomer activation. Similar results were obtained with other activation groups, e.g., 1-methyladenine, the type of activating groups defining whether an initiator is necessary and the length of the incubation period required to polymerize long RNA analogs [79]. 1-methyladenine is the sole activation group to support efficient and comparable incorporation of all four canonical nucleotides into polymers.

The montmorillonite-supported polymerization was found to be selective in terms of the product sequence and regiochemistry of linkages [69,80]. The elongation of an oligomer is determined by the nature of its 3-terminal nucleobase (purine or pyrimidine) and its regiospecificity: a 3′-5′-linked terminal nucleotide elongates more efficiently. Thus, the montmorillonite products will more likely be 3′-5′ isomers compared to that in a homogeneous aqueous medium where the 2′-5′ linkage is predominant. A 3′-terminal purine has a higher intrinsic reactivity, which leads to the preferential formation of purine-rich oligomers when mixtures of monomers are used. The last observation might be related to the more extensive stacking of the purine monomers.

The detailed association mechanism between monomers and clay surfaces and the polymerization mechanism itself was long unclear, as seemingly contradictory sets of results could not be explained in a consistent manner. Early results clearly indicated that the charges on the clay surface and on the monomers and products (the longer they were, the better their absorption) were essential for the association either through direct interactions, as the results with 1-methyladenine (a positively-charged, large heterocycle) seemed to indicate, or over bridging divalent metal ions (Figure 3A) [81]. However, more detailed investigations of the pH dependence of the association and polymerization have led to the proposal of an acid/base mechanism for both processes [82], although large concentrations of magnesium ions were present in the reaction dispersion.

TD polymerization on mineral surfaces was only superficially investigated on hydroxyapatite [83,84] and ferric oxide [85], and RNA fragments of lengths beyond those obtained in the absence of mineral were detected in the case of poly C and 2-methylimidazole G [84]. Moreover, the self-condensation products obtained on montmorillonite successfully served as templates for the synthesis of complementary strands [86] in a bulk aqueous medium.

Porous minerals, formed by the precipitation of ions in ion-rich hot water in contact with colder water at hydrothermal vents [71], could have also provided an environment conducive to prebiotic syntheses in general and RNA polymerization in particular. In this case, the mechanism leading to the RNA synthesis would have not been defined by the propensity of reactants to directly interact with the mineral surfaces, but rather by the fact that thermal gradients around the pores or clefts would have driven two intertwined processes: (a) molecules would have been subjected to laminar thermal convection parallel to the pore axis and (b) to thermophoresis along the temperature gradient (Figure 3B) [71]. In laboratory experiments with sub-micrometer pores, a gradient of 30 K per 100 µm had to be applied to lead to large accumulations of monomers or oligomers, as well as other molecules, such as fatty acids, to such an extent that they could form vesicles [87]. That is, the resulting concentration of fatty acids had exceeded the critical vesicle concentration. However, simulation work has shown that, given the right pore length, shallower gradients (down to 100 K over 1 m) could have been sufficient to reach similar or even larger enrichments [88]. Although direct evidence for a monomer self-condensation or a TD polymerization is still missing, these results are promising, as they demonstrate the possibility to concentrate small molecules (monomers or short oligomers) within a limited volume. In addition, the temperature gradient could also promote cycles of hybridization/dehybridization [89] that are essential for the amplification of RNA sequences. However, some results also show that high ionic strength media may lower the accumulation potential of pores/cleft models [87].

#### 4.1.2. Eutectic Phases

Eutectic phases in water/ice [73] and high salt/water [90] systems have also been proposed and could be relevant to the origin(s) of life assuming two different, but seemingly plausible early Earth environments: a cold Earth, fully covered by ice or with ice deposits, due to low sun irradiation [91,92] or a warm planet with hydrothermal fields [93]. In the former case, upon lowering the temperature of a reaction sample past its freezing point, water begins to nucleate and form pure water ice, which effectively reduces the volume of the remaining liquid that forms a network of brines (channels containing liquid water). As evermore water crystallizes, the solutes (activated monomers and metal ion catalysts) present in the sample are concentrated in these brines (Figure 3C). Further cooling to the so-called eutectic temperature (e.g., −21.1 °C for NaCl, [94]) will cause the remaining solution to solidify as the solutes reach their maximum solubility and precipitate. The final system characteristics, such as the volume of the eutectic phase, will depend on the final temperature and initial solute concentrations, monomers, metal ion catalysts and any other solutes present in the original solution.

In the salt/water eutectic phase system, the progressive evaporation of the solution containing monomers (NMPs) and inorganic salts at moderate temperatures (85 °C) leads to the crystallization of the salts concentrating the monomers within the remaining aqueous volume [90]. The inorganic salts are not playing the roles of catalyst per se, as heat is used. However, in contrast to the water/ice systems, efficient polymerization requires multiple cycles of drying followed by a rehydration of the sample. In both cases, microscopic investigations by epifluorescence of the eutectic systems [73,90] show that the fluorescent solutes were up-concentrated in between solid crystalline phases. Such a 3D organization resembles that proposed for the porous mineral (Figure 3B).

Both approaches have delivered RNA analog products in relatively high monomer equivalent yields (over 60% monomer incorporation) even during self-condensation of monomer mixtures [90,95]. In the case of the water/ice system, the detailed analysis of the product sequences revealed that the incorporation of both purines and pyrimidines usually corresponded to the original monomer composition of the samples [95]. The product lengths attained in the salt/water system were apparently longer than those in the ice/water case after shorter incubation times. Unfortunately, the evidence provided (gel patterns and a preliminary nanopore analysis versus sequencing gels and HPLC) does not allow for a definite length comparison [90]. Monomer self-condensation in the ice/water system was even detected in the presence of dipeptides acting as catalysts [96]. The catalytic activity of a non-coded, short peptide (Ser-His) is intriguing as it seems to support the idea of the co-evolution of the principal biologically-relevant polymers and of their functions.

TD polymerization was only demonstrated in the water/ice system. Interestingly, the initial elongation rates of RNA hairpin (i.e., a self-priming, self-templating RNA) studied as a function of the monomer concentration were comparable for all canonical nucleotides with optimal concentrations lying between 100 s µM and less than 2 mM [97]. The analysis of the sequence fidelity during TD polymerization was found to be rather high, and even in the presence of monomer mixtures, the main products (i.e., considering the fact that 2′-5′ and 3′-5′ linkages could be formed) after a 3-nucleotide elongation were complementary to the template sequences even though they contained residue sequences that blocked the polymerization in a bulk aqueous medium (see Section 3.2) [98].

Besides the potential advantages of eutectic phases (concentrating power, promotion of the non-covalent interactions between monomers (stacking and H-bonding) and catalysts), several authors mention a possible pre-organization of monomers and interactions with the extended ice-surfaces, which could also be hypothesized for the salt/water eutectic. Such effects have yet to be demonstrated. However, it is clear at least for the water/ice eutectic that its 3D organization significantly affects the outcome of the polymerization. The nature of the solutes and their concentration, even those that are not reactants and do not act as catalysts, will influence reactions occurring in the eutectic phase and its characteristic [74]. For example, the ionic strength of the solution containing the monomers will influence the efficiency of the polymerization [99]. Even the type of anions in the salt used to provide magnesium cations will affect the eutectic temperature by as much as 30 °C and impact the width of the aqueous channels, as shown for magnesium chloride and magnesium sulfate [74].

The eutectic phase in water/ice has also been shown to promote additional processes that are relevant to the RNA world hypothesis, such as the catalytic activity of ribozymes among other ligases [100,101] and polymerases [74,102], or even permits their self-assembly [103].

#### 4.1.3. Self-Assembled Molecular Systems

Contrary to previous heterogeneous media, self-assembled molecular systems require prebiotic synthesis of compounds, e.g., peptides [104], sugars [105] and amphiphiles [106], which given the right conditions, self-assemble into 3D structures that offer surfaces and specific volumes, i.e., several types of heterogeneous media. Amphiphile structures, such as bilayered vesicles, can preserve their characteristic molecular organization (Figure 3D) while being subjected to cycles of dehydration/rehydration [107] and are therefore able to capture, concentrate and order solutes between the bilayer stacks formed during dehydration. Cycles of dehydration/rehydration at relatively moderate temperatures, i.e., temperatures that do not promote the decomposition of organics [108], could have occurred in geothermal fields [75].

The promotion of RNA monomers’ self-condensation and DNA monomer TD polymerization was investigated [109,110] using acidic suspensions of 5′-phosphate monomers (free acid form) and various phospholipid vesicles (phosphatidylcholine and phosphatidic acids) that were heated to dryness under a flow of carbon dioxide, resulting in the formation of mixed nucleic acid compound/lipid-bilayer films. After incubation for up to 2 h at temperatures between 70 and 90 °C, the samples were rehydrated. Efficient polymerization could be observed after several drying-heating-rehydrating cycles (6 to 7 were optimal), as RNA analogs with up to 100 monomer units [108] were detected in sharp contrast to observations made in the absence of phospholipids. 

However, polymerization yields remained quite low (ca. 6% by weight). Interestingly, the length of the longest products depended on the number of different monomers present, as mixtures of two monomers (5′ UMP and AMP) yielded shorter polymers up to a length of 30 to 40 mers. The predominance of 2′-5′ linkages in the products can be surmised from the lack of product enzymatic digestion.

DNA monomer TD polymerization has also been reported in amphiphile matrices [110] under conditions where no product could be detected in absence of a template: a 61-mer heteropolymeric template designed to obtain the best possible polymerization was replicated in low yields. The polymerization yields on this heteropolymeric template were very low, and a complete copying of the template did not occur: the polymerization preferentially started in the middle of the template, perhaps due to the already-mentioned reduction of the polymerization at the template ends. Finally, the sequencing of the products after PCR amplification showed that only one out every 10 nucleobases was misincorporated, a frequency that should be compatible with information preservation while allowing for evolution.

Based on the reaction conditions and on the observations made by X-ray [107], a polymerization mechanism has been proposed based on an acid/base condensation reaction followed by the evaporation of the water byproduct, heat being therefore the driving force of the reaction. The lipid bilayer organizing power is also supported by an X-ray scattering study [107], where the distance between the 5′-phosphate of AMP and the 3′-OH of the ribose was estimated to be only of ~2.1 Å (the approximate length of a P-O bond), thus favoring the condensation reaction during dehydration.

The temperature at which the dehydration proceeds, while being moderate compared to those at marine vents in acidic conditions, could still reduce the polymerization yields, as the report of product depurination [111] and hydrolysis [112] confirmed it. The observed hydrolysis however did not reach that observed in bulk aqueous experiments [108]. Even though RNA product hydrolysis was clearly detected after four cycles of dehydration/rehydration [112], the hydrolysis products should be less detrimental to the formation of RNA, as it produces 3′or 2′ phosphate or 2′,3′cyclic monomers/oligomers and 5′-hydroxyl terminated oligomers, all of which could in principle serve as reactants for a new round of polymerization.

As for the other heterogeneous media, the lipid matrices could also have played some significant, additional roles in the emergence of compartmentalized precellular systems or protocells (the nucleic acid products can be encapsulated in the reforming vesicles during rehydration) [75,113] or even in the selection of nucleobases from the “prebiotic soup” [114].

## 5. Relevance of the Various Approaches to the Non-Enzymatic RNA Polymerization

The research undertaken to better understand the synthetic processes related to the emergence of the RNA world has significantly progressed in the last couple of years. In particular, several sets of “prebiotically-plausible” conditions have been found, which support the synthesis of RNA monomers, their efficient concentrating out of bulk aqueous solutions, their polymerization (self-condensation) and the replication of RNA sequences (TD polymerization). Interestingly, many of these processes require some form of “compartmentalization” that can be provided by several types of heterogeneous media. The relevance of any heterogeneous medium can be debated, as their basic properties (type of architecture, chemical composition, temperature and environmental conditions conducive to their formation) are derived from proposed and argued about scenarios for the early Earth environment. The similarities and complementarities between the various heterogeneous media cannot be overlooked, as most heterogeneous systems not only concentrate molecules out of the bulk aqueous solution, but additionally seem to promote their organization into molecular assemblies that are conducive to RNA polymerization. To some extent, they all protect both reactants and products against degradation. These various heterogeneous media are also complementary as far as they cover a large spectrum of environmental conditions that could have been present on the early Earth.

In the exploration of this type of heterogeneous catalysis, the composition of the samples studied has gradually become more complex. Mixtures of monomers and catalysts (metal-ions and small peptides), but also of various types of monomers/bystander molecules, from the point-of-view of their biochemical involvement in RNA polymerization, such polycyclic molecules [115], as well as of monomers/organic aggregates or solid particles (lipids, peptide and minerals) have been used. Interestingly, the results that were partially unexpected demonstrate the need to abandon the “pure” or “purified” system approach. Finally, the heterogeneous media can influence other crucial processes for RNA emergence and evolution, e.g., the selection of specific RNA sequences or molecular precursors, as well as foster catalytic properties. However, the new approach has also come at a cost: the analysis of these systems is becoming more complex, as well.

## 6. Conclusions

The long-standing problem of the RNA world hypothesis, i.e., the formation of polymeric RNA catalysts and their replication/amplification, has clearly not yet been resolved as the demonstration of the emergence of any catalytically-active RNA from monomers is still elusive. However, many potential environments that are plausible on the early Earth have been shown to promote some processes deemed essential for the emergence of an RNA world or its precursors. At this time, the evidence available about the early Earth conditions, as well as the fragmentary nature of the collected data preclude a definitive resolution of the question, but it is also conceivable that several heterogeneous media may have played a role either simultaneously (assuming an early Earth with various microenvironments) or at different stages of the RNA world emergence and evolution. Nonetheless, the most recent advancements in this research field have clearly highlighted the need for a systemic approach to the question that includes geochemistry, geophysics and more complex chemical systems (in opposition to the purified systems often used early on) to explore potential synergies between various biopolymers or their precursors, as well as other chemicals present in the prebiotic environment.
